# A Historical Perspective on Presentations of Hypertensive Acute Heart Failure

**DOI:** 10.4172/2329-9517.1000275

**Published:** 2017-05-15

**Authors:** Chad E Darling, Jiaoyuan Elisabeth Sun, Jordan Goldberg, Peter Pang, Christopher W Baugh, Darleen Lessard, David D McManus

**Affiliations:** 1 Department of Emergency Medicine, University of Massachusetts Medical School, Worcester, MA, USA; 2 Department of Quantitative Health Sciences, University of Massachusetts Medical School, Worcester, MA, USA; 3 Department of Medicine, Division of Cardiology, University of Massachusetts Medical School, Worcester, MA, USA; 4 Department of Emergency Medicine, University of Indiana, Indianapolis, IN, USA; 5 Department of Emergency Medicine, Brigham and Women’s Hospital, Boston, MA, USA

**Keywords:** Acute heart failure, Systolic blood pressure, Emergency department, Epidemiology

## Abstract

**Background:**

The initial systolic blood pressure (SBP) in patients presenting to the hospital with acute heart failure (AHF) informs prognosis, diagnosis, and guides initial treatment. However, over time AHF presentations with elevated SBP appear to have declined. The present study examined whether the frequency of AHF presentations with systolic hypertension (SBP >160 mmHg) declined over a nearly two-decade time interval.

**Methods:**

This study compares four historical, cross-sectional cohorts with AHF who were admitted to tertiary care medical centres in the North-eastern USA in 1995, 2000, 2006, and 2011–13. The main outcome was the proportion of AHF patients presenting with an initial SBP >160 mmHg.

**Results:**

2,366 patients comprised the study sample. The average age was 77 years, 55% were female, 94% white, and 75% had prior heart failure. In 1995, 34% of AHF patients presented with an initial SBP >160 mmHg compared to 20% in 2011–2013 (p<0.01). Multivariate logistic regression demonstrated reduced odds of presenting with a SBP >160 mmHg in 2006 (0.64, 95% CI 0.42–0.96) and 2011–13 (0.46, 95% CI 0.28–0.74) compared with patients in 1995.

**Conclusion:**

The proportion of patients with AHF and initial SBP >160 mmHg significantly declined over the study time period. There are several potential reasons for this observation and these findings highlight the need for ongoing surveillance of patients with AHF as changing clinical characteristics can impact early treatment decisions.

## Introduction

Worldwide, over 20 million people have chronic heart failure (HF) [1]. Patients with HF are prone to recurrent acute HF (AHF) exacerbations, and in the United States the majority of such patients are cared for in an emergency department (ED). Acute HF presentations account for nearly one million visits [2] and hospital admissions annually in the U.S. alone [3,4]. Efficiently diagnosing AHF and initiating appropriate therapies for patients with AHF can be challenging since multi-morbidity is common in patients with HF. However, several clinical parameters available at the time of hospital presentation, including history, exam, lab testing, xray, EKG, and point of care ultrasound, provide information that is useful in diagnosing the presence of AHF and guiding treatment [5]. Expert opinion has suggested that the initial, ED management of patients with AHF be based on the patient’s clinical profile [6]. This is, in part, influenced by vital signs at the time of presentation and in particular presenting blood pressure (BP) [7,8].

Historically the majority of patients with AHF present to the hospital with either elevated or normal blood pressure (BP) [7–9]. These initial BP values can be used to classify patients as having either “vascular failure,” as defined by a systolic blood pressure (SBP) of >160 mmHg, or “cardiac failure,” as defined by a normal or low SBP (e.g., <150 mmHg) [9,10]. Although far from an exact science, presenting blood pressure profile is related to type of HF, with patients having preserved ejection fraction (HFpEF) more commonly presenting with an elevated SBP [5,9]. Since treatment decisions about AHF must frequently be made in the ED without echocardiographic data, SBP represents a potential initial guide for early AHF management.

Epidemiological studies suggest that the clinical characteristics, management, and prognosis of patients with HF have changed over the past several decades. Compared to prior years, contemporary patients presenting with AHF are older, have a greater comorbidity burden, and have increased short and longer-term survival [11]. Although the prognosis from HF has improved over time, rates of hospital admission for HF are not in decline, suggesting that AHF remains a common and costly condition. Between the 1990s and the mid-2000s it appeared that there was a decline in the number of patients presenting with AHF and elevated systolic hypertension on admission. We undertook the present study to examine whether there was indeed a historical change in the frequency of AHF presentations with systolic hypertension (SBP >160 mmHg) on arrival to the ED in 4 historical, cross-sectional, cohorts between 1995 and 2013.

## Methods

### Study population sources

We derived our study population from data collected on patients seen in the EDs of 3 teaching hospitals in Worcester, MA (USA) and subsequently admitted to the hospital with a diagnosis of AHF. Two National Institute of Health sponsored research projects, conducted by members of the same research group, contributed to the database for the present study. The first study was the Worcester heart failure study (WHFS) which was a large, multi-center investigation that documented the clinical characteristics, treatments, and outcomes of adult patients admitted with AHF to all medical centers in central Massachusetts over multiple study years between 1995 and 2006 [11,12]. The second study was an ED-based extension of the WHFS using similar methodology and was conducted from 2011–2013. Both studies conform with the principles outlined in the Declaration of Helsinki and underwent full review by the Committee for the Protection of Human Subjects in Research at the University of Massachusetts Medical School. All study activities described below were approved, and all subjects either signed a written informed consent document prior to participation or were considered exempt.

## Additional Study Details

### The Worcester heart failure study

The WHFS collected data on adult patients admitted to multiple (n=11) hospitals in central Massachusetts (MA) over several study years between 1995 and 2006 [11,12]. For the present study, we restricted the WHFS sample to patients admitted in 1995, 2000, and 2006 to three teaching hospitals in Worcester, MA. Patients in the WHFS were identified by trained study staff who performed a standardized review of the medical records of patients with primary and/or secondary International Classification of Diseases (9th Revision) diagnoses consistent with a possible admission for AHF. Patients who were discharged with the HF code of (428.XX) were used as the primary means for identifying cases of possible AHF. Subsequently only cases that met the Framingham criteria for HF were included in the final dataset [13].

### ED-based study

The ED-based, observational, investigation began in September 2011, and enrolled patients over 2 years from same teaching hospitals included in the WHFS. Patients were identified and initially enrolled in the ED by trained study staff based on history, physical exam, physician impression, and lab values consistent with the occurrence of possible AHF. The HF diagnosis was confirmed (or refuted) by subsequent chart review based on whether the subject met Framingham criteria for AHF as in the WHFS.

### Inclusion and exclusion criteria

Only adult (>18) patients admitted to one of the study teaching hospitals after being evaluated in the ED were included for both the WHFS and ED enrolled patients. As mentioned, our diagnostic criterion for AHF was the Framingham criteria which were chosen because it has been used in several studies, including the WHFS, [14,15] and is straightforward to apply. These criteria include the presence of 2 major (e.g. radiological evidence of pulmonary edema) or 1 major and 2 minor (e.g. extremity oedema, exertional dyspnoea) criteria [13]. Subjects were excluded if they had hypotension (SBP <95 mmHg) or cardiogenic shock, or if they were on haemodialysis.

### Data collection activities

Both contributing studies collected the majority of their data from structured chart reviews. Data included patients’ demographic characteristics, past medical history, physical exam findings, vital signs, home medications, clinical characteristics, admission status (e.g., ICU versus floor), and laboratory results. For the main variable of interest, SBP, we used the first available reading obtained after the patient arrived at the hospital ED. The chart review also obtained information on short-term clinical outcomes including inpatient length of stay (LOS), need for ICU admission, endotracheal intubation, complications, and ejection fraction findings.

## Data Analysis

### Methods used to summarize the data

**Figure 1:**
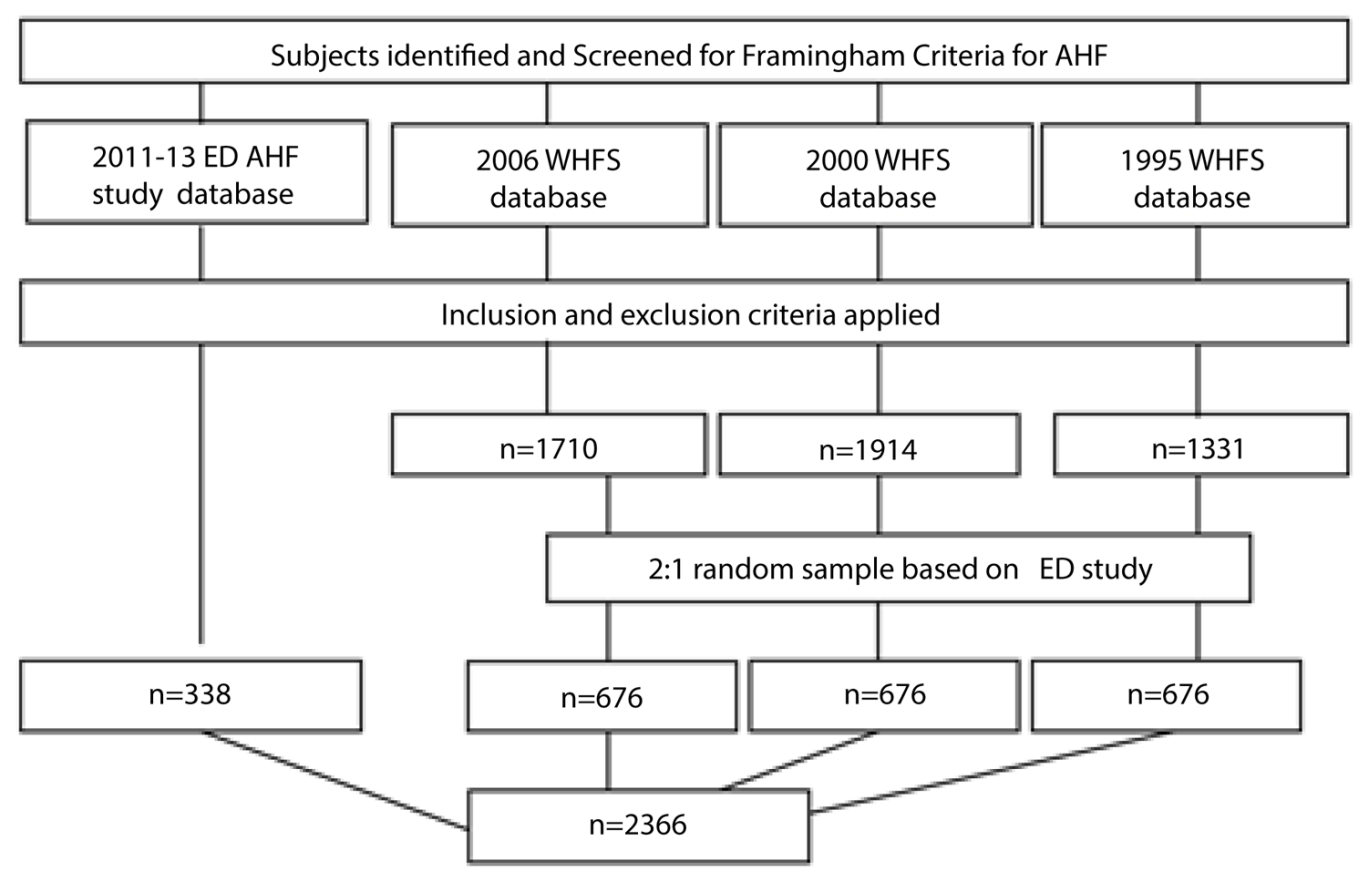
Diagram of subject enrolment.

A total of 338 subjects were available from the ED-based AHF study for analysis. To increase statistical power, we took a 2:1 random sample of subjects from each WHFS year (compared to the ED-based study) which resulted in a total of 676 patients in each WHFS cohort (1995, 2000, 2006) (Figure 1). Differences in the demographic and clinical characteristics of patients in each of the four study groups were compared by chi-square tests for categorical and ANOVA for continuous variables (SAS, Cary, NC).

### Methods used to test the principal study hypothesis

Our primary study endpoint was the percentage of patients presenting to the ED with AHF who had a SBP >160 mmHg during each of the years under study. Our hypothesis was that the frequency of AHF presentations accompanied by hypertension (SBP >160 mmHg) had declined over the study interval. We used a SBP cutoff of >160 mmHg because it was the highest BP category defined in the OPTIMIZE-HF registry study [9]. In published guidelines it identifies patients with AHF who are in a low risk group, [16] and also because this group would be most likely to require acute treatment to lower their BP in the ED. For the univariate analyses, differences in the average SBP at the time of ED presentation according to study year were compared by chi-square tests of statistical significance. In addition, a multivariate logistic regression analysis was performed to examine whether study year was associated with a decrease in hypertensive AHF presentations over time at a SBP cut point of 160 mmHg. We set 1995 as the referent year and created a multiple logistic regression model by adding potential confounding variables to the analysis based on clinical relevance and a p value of <0.20 on univariate examination. These factors included age, sex, race, body mass index, hematocrit, outpatient medications, a history of hypertension, atrial fibrillation, diabetes mellitus, kidney disease, and hypercholesterolemia. We also considered additional confounding factors of prognostic importance that have been shown to be associated with mortality and other clinical outcomes in patients with AHF [17,18]. Of note, EF findings were missing in 58% of the study sample and were not controlled for in the regression models. Similarly, B-type natriuretic peptide (BNP) levels were not available in the 1995 and 2000 study cohorts and were not included in our regression analyses.

## Results

### Characteristics of the entire study population

A total of 2,366 individuals admitted with AHF comprised our study population (Figure 1). The average age of this population was 77 years, 55% were female, 92% were white, and 75% had been previously diagnosed with HF. Between 1995 and 2011–13 there were several notable changes in the clinical characteristics of these patients. Compared to patients admitted in 1995, patients in the more recent study years of 2011–13 were significantly more likely to have a history of hypertension, atrial fibrillation, chronic kidney disease, and hyperlipidemia. These patients were also more likely to be taking ACE-Inhibitors and beta-blockers and have a shorter length of hospital stay, but were less likely to be taking digoxin than patients in 1995 (Table 1).

### Trends in the characteristics of patients with SBP >160 mmHg

Among patients with AHF and a SBP >160 mmHg, the average age was 76 years, 61% were female, and 91% were white. Consistent with the findings in the entire study sample, between 1995 and 2011–13 the characteristics of patients with AHF and a presenting SBP >160 mmHg also changed. In more recent study years, AHF patients with systolic hypertension on presentation were less likely to be women, more likely to have a shorter LOS and a lower mean SBP on admission, and fewer were prescribed digoxin (Table 2).

### Mean SBP and the frequency of elevated SBP over time

**Figure 2:**
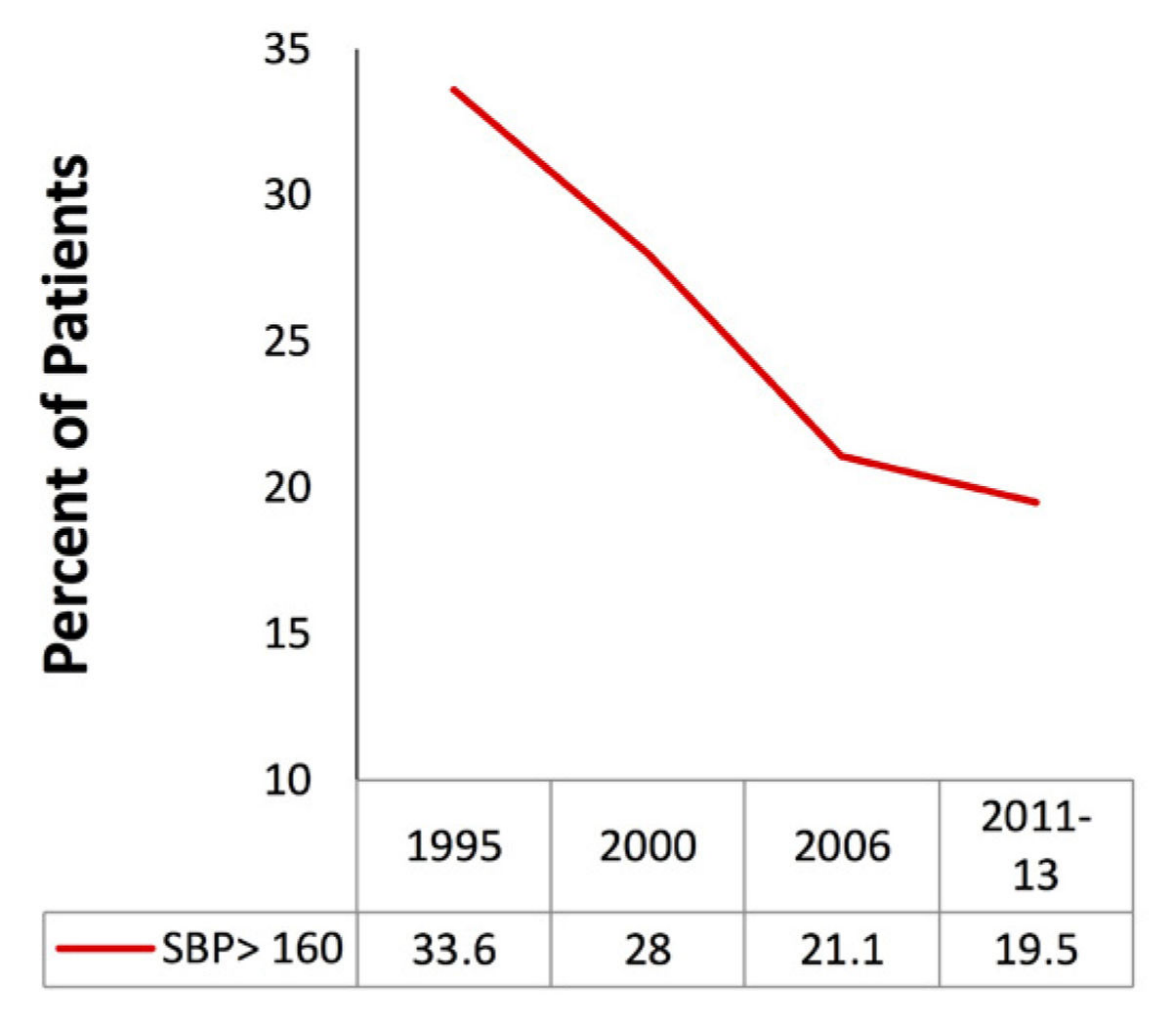
Percent of patients with AHF presenting with systolic blood pressure >160 mHg over time.

The SBP at presentation averaged 151 mmHg in 1995 and decreased over time to 140 mmHg by 2011–2013 (p<0.01). In 1995, 33.6% of all AHF patients had a SBP>160 mmHg which decreased to 19.5% by 2011–2013 (p<0.01) (Figure 2). 45% of patients had ejection fraction (EF) measured during their index inpatient admission. For the subset of patients with EF measurements, SBP trends were analyzed by either preserved (≥50%) or reduced (≤40%) EF. In patients with reduced EF, average SBP declined from 152 mmHg in 1995 to 138 mmHg in 2011–13 (p=0.03). However, for those patients with preserved EF SBP declines were much less significant and averaged 156 mmHg in 1995 and 149 mmHg in 2011–13.

**Figure 3:**
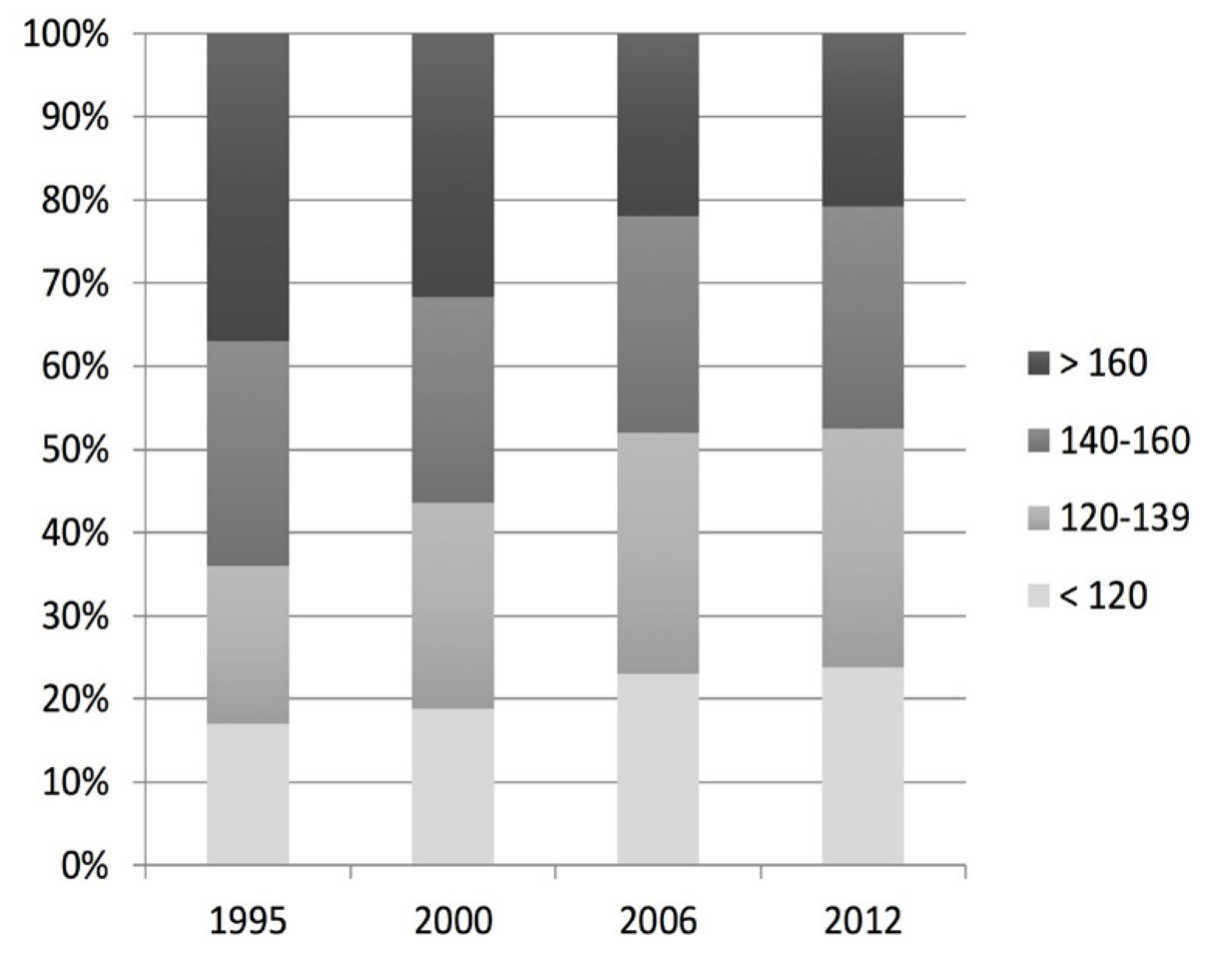
Distribution of systolic blood pressure (mmHg) by study year.

The distribution of SBP across four BP strata has also changed over time. Patients hospitalized with AHF during more recent years (after 2006) demonstrated a more balanced and even distribution of SBP than earlier years where a greater percentage of patients fell into the higher (>140 mmHg and >160 mmHg) SBP strata (Figure 3).

When compared to patients hospitalized in 1995 (referent year), the crude odds ratios demonstrated that there was a significantly lower odds of patients with AHF presenting with SBP >160 mmHg in 2000, 2006, and 2011–2013. After adjustment for several demographic, medical history, and clinical factors, only the 2006 and 2011–13 cohorts were found to have a significantly reduced odds of presenting with a SBP >160 mmHg (Table 3).

## Discussion

In the present study we compared the frequency of patients with AHF presenting to the ED with SBP greater than 160mmHg in four cross-sectional study cohorts between 1995 and 2011–13. The rationale for studying presenting SBP at presentation for patients with AHF is to try to understand if there was a change in the epidemiology of these patients over time and because of expert recommendations to use SBP as a guide to early diagnostic and treatment approaches. Our results demonstrate that there was a significant decrease over our study time period in the proportion of AHF patients who present with SBP >160 mmHg, as well as a similar decrease in the mean SBP over greater than 15 years beginning in 1995.

Despite a decrease in the proportion of patients with an SBP >160 mmHg over time, the overall mean SBP results we observed are consistent with other observational studies of patients with AHF conducted in the same time period. The acute decompensated heart failure national registry (ADHERE) collected data on patients with AHF who were admitted to 263 medical centers from all regions of the United States between 2001 and 2006. In ADHERE, patients admitted between 2001 and 2003 were found to have a mean initial SBP of 144 mmHg which falls between our mean SBP’s for 2000 and 2006 which were 147 mmHg and 141 mmHg respectively [19]. Similarly, the organized program to initiate lifesaving treatment in hospitalized patients with heart failure (OPTIMIZE-HF) national registry found that the average SBP of patients admitted with AHF in 2003 and 2004 was 143 mmHg. Although we found a decline in the initial SBP of patients with AHF over our study time-period it is important to note that most still had normal to elevated SBP at the time of hospital arrival. Our results also suggest that patients with preserved ejection fraction were the least affected by this overall trend of reduced initial SBP over time.

The importance of SBP in providing a framework for clinicians to clinically categorize and better manage patients with AHF has been discussed in several studies [5,7,8]. The OPTIMIZE-HF registry of nearly 50,000 patients found that patients with higher SBP’s were more likely to be female, African American, and to have heart failure with a preserved ejection fraction [9]. OPTIMIZE-HF also documented the relationship between initial SBP and short-term (inpatient) and post discharge prognosis, where patients having higher SBPs (>140 mmHg) were found to have a decreased mortality compared with patients in lower SBP strata [9]. Lastly, an American Heart Association working group focusing on the diagnosis and treatment of AHF in the ED setting has advocated an approach whereby SBP is central to early risk stratification with patients having a SBP>160 mmHg falling into the lowest risk prognostic group [16]. Moreover, prior literature has characterized patients with AHF and significantly elevated SBP as having “vascular failure” rather than “cardiac failure”. This distinction is helpful to clinicians as they attempt to distinguish between patients with different treatment needs [8,20]. The vascular failure patient typically does not have significant total body fluid overload and, as a result, early treatment focuses on afterload reduction rather than intensive diuresis [8,20].

It is unclear why the proportion of patients with AHF and hypertension underwent the historical decline we observed. However, our data demonstrates that many demographic and clinical features of these patients have also changed during the last few decades. Since 1995, patients enrolled in studies of AHF in our geographic area have become older, have a greater comorbid disease burden, but have an improved post discharge prognosis [21]. Similarly, other studies have documented improvement in the long-term survival of patients discharged from the hospital after AHF treatment [22,23]. A large North American study of more than 55 million Medicare patients discharged with a diagnosis of AHF found that admission rates for AHF declined significantly between 1998 and 2008, perhaps reflecting improved management of these patients [3]. Overall these changes may partly explain our results.

However, other factors may also explain our findings. First, the outpatient management of heart failure changed significantly while our data was collected [24,25]. There is also increased recognition that outpatient hypertension management in HF patients can decrease the incidence rate of AHF presentations [26]. In our sample there was a steady increase in the use of ACE-Inhibitors and beta-blockers in isolation and in combination over time. Others have documented similar increases in the use of these medications over time [27]. It is unclear how the outpatient management of patients with HF influences the SBP when a patient presents with AHF, but it may, in part, explain the trends we observed.

Lastly, the management of hypertension has changed significantly with new definitions of hypertension and targets for therapy proposed with each Joint National Committee on the Detection, Evaluation, and Treatment of High Blood Pressure (JNC) update since 1977 [28]. In our study, we found a significant increase in the proportion of patients with pre-existing hypertension over time which likely reflects the increased comorbid disease burden in these patients, the changing definitions of hypertension, and the heightened focus on this disease. Since hypertension is an important cause of heart failure, and blood pressure control has improved over time, these changing trends may have influenced our results [28,29].

### Study Strengths and Limitations

The major strength of this study lies in its ability to combine detailed current and historical data in order to better understand how the presentation of patients with AHF may have changed over time. However, there are several limitations that should be considered when interpreting our findings. First, the WHFS data were compared to an ED-based AHF study and there were some differences in how the patients were identified. Such heterogeneity could introduce a potential selection bias. However, the significant trend in reduction of hypertensive presentations in patients with AHF was also observed when only considering the most recent WHFS cohort (2006) (excluding ED-based 2011–13 data), reinforcing the likelihood of our observed trends being real. Secondly there was no standard method by which the patient’s initial SBP was measured although this limitation would have been consistent across all study years. Third, ejection fraction (EF) is an important variable that is related to presenting SBP, but data on EF were missing in a large proportion of subjects and was not included in our regression models. Similarly, BNP was not obtained in the first 2 patient cohorts due to it not being used at all the study sites. Changes in prehospital treatment may also have influenced the initial presenting SBP’s, but the proportion of patients with AHF transported by ambulance has not significantly changed over time (data not shown). Patient with a SBP <95 mmHg was excluded from both studies due to the initial goals of the ED-based research protocol. This could result in the mean SBP’s we report to be somewhat higher than values reported in other studies. This study was also conducted in a single region in the USA, which may limit the generalizability of our findings to other geographic areas where the racial, socio-demographic, and clinical characteristics of patients differ.

## Conclusion

Our study demonstrates that the proportion of patients with AHF accompanied by a SBP >160 mmHg at the time of presentation to the hospital declined significantly between 1995 and the late 2000s. In addition, the mean SBP at presentation also declined over the nearly 2 decade period covered under this study. Why these changes have occurred is unknown, but they are likely a result of the changes that have occurred in the outpatient management of both heart failure and hypertension over the last 15–20 years. It also highlights the need for ongoing surveillance studies of patients with AHF as the risk of HF increases in prevalence with the aging of the population.

## Figures and Tables

**Table 1:** Selected demographic and clinical characteristics of the entire study sample.

	Study Year				
	1995	2000	2006	2011–13	p-value
Demographics	(n=676)	(n=676)	(n=676)	(n=338)	
Age (mean, yrs)	76.7	76.9	77.6	74.8	<0.01
Female (%)	57.9	56.7	56.4	41.2	<0.01
White (%)	95.9	91.4	90.1	89.6	<0.01
Body mass index (kg/m^2^)	26.9	27.7	28.0	31.7	<0.01
**Medical/Surgical History (%)**					
Hypertension	64.4	68.1	82.7	89.4	<0.01
Atrial fibrillation	36.7	36.7	45.9	53.9	<0.01
Diabetes mellitus	39.5	38.2	37.9	52.7	<0.01
Coronary heart disease	56.7	58.4	58.1	58.3	0.6
Chronic pulmonary disease	38.0	34.6	40.1	35.8	0.6
Stroke	15.8	16.4	14.1	18.6	0.9
Chronic kidney disease	20.6	22.0	36.5	46.5	<0.01
Heart failure	75.3	73.3	79.1	71.9	0.6
Hypercholesterolemia	14.4	24.9	53.3	73.7	<0.01
Mean EF (%)	40.7	43.2	47.3	44.4	0.51*
**Home Medications (%)**					
ACE-Inhibitors	39.1	36.1	39.6	55.9	<0.01
Beta Mockers	21.3	40.4	64.4	74.9	<0.01
ACE-I + Beta Stockers	7.0	17.5	28.7	44.4	<0.01
Calcium Channel Blockers	33.6	24.0	23.8	26.3	<0.01
Diuretics (any)	70.4	66.7	72.0	77.5	<0.01
Digoxin	39.9	33.1	18.1	18.3	<0.01
**Laboratory findings**					
Creatinine	1.5	1.5	1.5	1.6	0.5
Hematocrit (%)	37.2	36.2	35.3	35.1	<0.01

# - missing data in >50% of patients, ACE-I= angiotensin converting enzymeinhibitor, Creatinine (mg/dl)

**Table 2:** Selected demographic and clinical characteristics of patients of patients with a SBP >160 mmHg on presentation.

	Study Year				
	1995	2000	2006	2011–13	p-value
Demographics	(n=227)	(n=189)	(n=143)	(n=66)	
Age (mean, yrs)	76.8	76.4	76.7	73.5	0.25
Female (%)	66.5	61.4	53.9	54.6	<0.01
White (%)	96	87.3	89.5	90	0.09
Body mass index (kg/m^2^)	27.9	28.6	28.3	33.4	<0.01
**Medical/Surgical History (%)**					
Hypertension	75.3	77.8	88.1	93.9	<0.01
Atrial fibrillation	32.6	29.6	30.8	45.5	0.23
Diabetes mellitus	44.9	42.3	39.9	50	0.96
Coronary heart disease	55.1	57.1	49	54.6	0.36
Chronic obstructive pulmonary disease	35.2	31.2	34.3	25.8	0.36
Stroke	18.1	12.1	16.8	25.8	0.27
Chronic kidney disease	19.8	22.8	37.8	42.4	<0.01
Heart failure	72.3	66.1	72	60.6	0.35
Hyperlipidemia	19.4	25.4	48.3	74.2	<0.01
**Home Medications (%)**					
ACE-Inhibitors	36.1	34.4	39.9	62.1	<0.01
Beta Blockers	26.9	48.2	62.9	77.3	<0.01
ACE-I + Beta Blockers	7.5	21.1	29.4	50	<0.01
Digoxin	36.6	26.5	14	7.6	<0.01
Diuretics (any)	64.8	56.6	60.1	69.7	0.81
**Laboratory findings**					
Creatinine, mean (mg/dl)	1.46	1.5	1.5	1.55	0.8
Hematocrit mean (%)	38.1	36.9	36.2	35.9	0.03

** data missing in 55% of cases

**Table 3:** Crude and adjusted odds ratios for patients presenting with a SBP >160 mmHg.

Year	Crude OR	Adjusted OR*
1995^0^	1	1
2000	0.7	0.94
	(0.54–0.90)$	(0.67–1.31)
2006	0.41	0.64
	(0.30–0.55)	(0.42–0.96)
2011–13	0.35	0.46
	(0.23–0.52)	(0.28–0.74)

* adjusted for the following variables: age, sex, race, body mass index, hypertension, atrial fibrillation, diabetes mellitus, kidney disease, hypercholesterolemia, outpatient medication (ace-inhibitors, beta-blockers, diuretics, digoxin, calcium channel blockers, hematocrit;

0 1995 is the referent year;

$ 95% CI

## Abbreviations

AHF: Acute Heart Failure
SBP: Systolic Blood Pressure
ED: Emergency Department
WHFS: Worcester Heart Failure Study
